# A Preclinical Rodent Model for Repetitive Subconcussive Head Impact Exposure in Contact Sport Athletes

**DOI:** 10.3389/fnbeh.2022.805124

**Published:** 2022-03-09

**Authors:** Brian D. Stemper, Alok Shah, Rachel Chiariello, Cassandra McCarthy, Kristin Jessen, Bailey Sarka, Jack Seifert, Matthew D. Budde, Kevin Wang, Christopher M. Olsen, Michael McCrea

**Affiliations:** ^1^Joint Department of Biomedical Engineering, Medical College of Wisconsin, Marquette University, Milwaukee, WI, United States; ^2^Department of Neurosurgery, Medical College of Wisconsin, Milwaukee, WI, United States; ^3^Clement J. Zablocki Veterans Affairs Medical Center, Milwaukee, WI, United States; ^4^Neuroscience Research Center, Medical College of Wisconsin, Milwaukee, WI, United States; ^5^Department of Pharmacology and Toxicology, Medical College of Wisconsin, Milwaukee, WI, United States; ^6^Gryphon Bio, Inc., South San Francisco, CA, United States

**Keywords:** traumatic brain injury, head acceleration, neurofilament light (NFL), microgliosis, Morris water maze (MWM), DAPI, cognition, Iba1

## Abstract

Repetitive subconcussive head impact exposure has been associated with clinical and MRI changes in some non-concussed contact sport athletes over the course of a season. However, analysis of human tolerance for repeated head impacts is complicated by concussion and head impact exposure history, genetics, and other personal factors. Therefore, the objective of the current study was to develop a rodent model for repetitive subconcussive head impact exposure that can be used to understand injury mechanisms and tolerance in the human. This study incorporated the Medical College of Wisconsin Rotational Injury Model to expose rats to multiple low-level head accelerations per day over a 4-week period. The peak magnitude of head accelerations were scaled from our prior human studies of contact sport athletes and the number of exposures per day were based on the median (moderate exposure) and 95th percentile (high exposure) number of exposures per day across the human sample. Following the exposure protocol, rats were assessed for cognitive deficits, emotional changes, blood serum levels of axonal injury biomarkers, and histopathological evidence of injury. High exposure rats demonstrated cognitive deficits and evidence of anxiety-like behaviors relative to shams. Moderate exposure rats did not demonstrate either of those behaviors. Similarly, high exposure rats had histopathological evidence of gliosis [i.e., elevated Iba1 intensity and glial fibrillary acidic protein (GFAP) volume relative to shams] in the basolateral amygdala and other areas. Blood serum levels of neurofilament light (NFL) demonstrated a dose response relationship with increasing numbers of low-level head acceleration exposures with a higher week-to-week rate of NFL increase for the high exposure group compared to the moderate exposure group. These findings demonstrate a cumulative effect of repeated low-level head accelerations and provide a model that can be used in future studies to better understand mechanisms and tolerance for brain injury resulting from repeated low-level head accelerations, with scalable biomechanics between the rat and human.

## Introduction

The understanding of biomechanical mechanisms for sport-related concussion continues to evolve as studies highlight the possible role of repetitive subconcussive head impact exposure for incident concussion and the development of concussion-like symptoms. The term subconcussive head impact exposure (HIE) refers to an athlete’s head impact burden during routine participation in contact sports in terms of the number, magnitude, and frequency of head impacts that do not individually result in concussion. Investigators hypothesized that consecutive head impacts sustained during participation in contact sports may contribute to decreasing mechanical tolerance for incident concussion, which could contribute to concussive injury following unremarkable head impacts as was reported previously (Duhaime et al., 2012; Beckwith et al., 2013; Stemper et al., 2019b). For example, concussion studies involving collegiate and high school contact sport athletes demonstrated greater HIE on the injury date, Beckwith et al. (2013) elevated HIE in concussed athletes relative to matched controls (Stemper et al., 2019b) and higher impact density (summed impact magnitude divided by the time between impacts for the 20 impacts leading up to concussion) in concussed athletes (Broglio et al., 2017). Despite the fact that concussive impacts are often unremarkable when compared to the severity of head impacts sustained across all athletes, the magnitudes are often remarkable for the individual athlete, with a majority ranking in the top five most severe head impacts sustained by that athlete in their season of injury (Rowson et al., 2018). This individualized concussive impact tolerance may be attributable, at least in part, to recent or within-season HIE. These studies imply a priming effect from HIE that may reduce tolerance and contribute to concussive injury from head impacts/accelerations that would normally be subconcussive.

Other studies provided evidence that HIE may influence the gradual onset of concussion-like symptoms (Talavage et al., 2016). That theory implies that some subconcussive head impacts contribute to subclinical changes that accumulate with continued HIE and eventually manifest as concussion-like symptoms in the athlete despite an absence of concussion report. Researchers provided evidence of clinical and MRI changes in non-concussed athletes with elevated HIE over the course of a single contact sport season. For example, imaging studies incorporating non-concussed football athletes identified significant associations between HIE metrics and measures of white matter integrity obtained using diffusion kurtosis imaging (DKI) (Davenport et al., 2016) or diffusion tensor imaging (DTI) (Bazarian et al., 2014; Davenport et al., 2014). Other studies reported cognitive changes in non-concussed football athletes that sustained elevated HIE during a single season of participation (McAllister et al., 2012; Talavage et al., 2014). A recent study also demonstrated a linear relationship between the number of head impacts over a season of ice hockey and cognitive processing measures quantified using changes in brain vital signs (Fickling et al., 2021). These associative findings provide evidence of progressive subclinical changes in contact sport athletes related to elevated levels of ongoing HIE sustained over the course of a single season. However, given the variability in season-long HIE (Stemper et al., 2020) and health/athletic history (Montenigro et al., 2017) between individuals, a causative relationship between specific levels of HIE and clinical outcomes has yet to be defined.

Preclinical models present the best opportunity to characterize a causative relationship between specific input levels of HIE and the resulting behavioral and pathological outcomes as input HIE can be specifically controlled, health/athletic history is removed as a variable, and outcome measures can be specifically targeted. This is particularly important for the study of repetitive head insults due to the significant variability in the number and severity of head impacts recorded in human studies. To date, a relationship between repetitive subconcussive exposures and behavioral/pathological outcomes has not been defined using a preclinical model. Some studies have focused on repeated head insults although insult magnitudes were not specifically focused in the subconcussive realm and exposure characteristics (i.e., number and frequency) were not based on human exposures. Preclinical studies reported dose-dependent relationships between the number of concussive exposures and behavioral, cognitive, and pathological outcomes (Shultz et al., 2012a; Prins et al., 2013). For example, three mild fluid percussion injuries produced short-term inflammation and five injuries produced more chronic inflammation combined with anxiety and depression behaviors (Shultz et al., 2012a). Other studies focused on repetitive “subconcussive” exposures (i.e., mild lateral fluid percussion or low level impact accelerations) that resulted in acute neuroinflammatory responses (Shultz et al., 2012b) pain hypersensitivity (Bree et al., 2020), and neuromotor dysfunction (Lavender et al., 2020). Based on the results of one study, the authors hypothesized that repeated “subconcussive” exposures could contribute to cumulative and neurodegenerative changes in the brain (Shultz et al., 2012b). Based on the findings of progressive behavioral and pathological changes associated with repeated insults and inflammatory changes associated with “subconcussive” exposures, the objective of the present study was to characterize behavioral and pathological changes associated with repeated subconcussive exposures using a rat head rotational acceleration model. The use of a rotational acceleration model was important as it enables experimental modeling of human contact sport exposures in terms of the number, frequency, and scaled magnitudes of recorded head impacts. Head impact exposure characteristics in this study were based on our prior human studies.

## Materials and Methods

All injury and behavioral assessment protocols used in this study were approved by the Institutional Animal Care and Use Committee (IACUC) at the Clement J. Zablocki Veterans Affairs Medical Center in Milwaukee, WI, United States.

### Experimental Groups

Male Sprague Dawley rats (342 ± 12 g; range: 306–371 g) were used to characterize the graded effects of repeated “subconcussive” head rotational accelerations in comparison to control rats receiving sham procedures and rats receiving a single high magnitude head rotational acceleration. A total of four experimental groups based on head acceleration exposures were included: high exposure, moderate exposure, single injury, and sham. Each group consisted of fifteen rats (total *n* = 60). The magnitude and frequency of head rotational accelerations for both exposure groups were based on our prior human studies of preseason head impact exposure in NCAA Division I football athletes (Stemper et al., 2019a). Specifically, rats in the high exposure group received 30 head accelerations per day, 5 days per week for 4 weeks (total 600 head accelerations). Thirty head accelerations per day represented the 95th percentile for the number of recorded head impacts per day from our human studies. Rats in the moderate exposure group received 8 head accelerations per day, 5 days per week for 4 weeks (total 160 head accelerations). Eight head accelerations per day represented the median number of recorded head impacts per day from our human studies. The magnitude of head accelerations was based on the median peak head rotational acceleration value for all recorded head impacts in our human studies, which was scaled to the rat (100 krad/s^2^) according to accepted brain mass scaling procedures (Fijalkowski et al., 2007). That magnitude was less than 50% of the peak rotational acceleration magnitude that did not produce any behavioral changes relative to shams in our prior research, Stemper et al. (2015) and was therefore considered to be a subconcussive exposure. Rats in the single injury group received a single high magnitude head rotational acceleration with peak magnitude of 450 krad/s^2^. Peak rotational acceleration magnitude for the single injury group was consistent with mild traumatic brain injury according to our prior studies (Stemper et al., 2015, 2016a). The single injury was performed on the Friday prior to the initiation of the behavioral protocol to provide consistent timing with the end of the exposure/sham protocols relative to behavioral assessments. Sham rats received the sham procedure, including anesthesia and analgesia, 5 days per week for 4 weeks.

### Head Rotational Acceleration Protocol

Rats were exposed to head rotational accelerations using the Medical College of Wisconsin (MCW) Rotational Injury Device (Stemper et al., 2016b). The experimental device produces graded levels of injury through head rotational acceleration and consists of two parts: a helmet and impacting system. The helmet fits securely on the rat head without the need for surgery or adhesive attachment and has a laterally extended moment arm. The impacting system consists of a launcher, drop tower, and impacting rod. The pneumatic launcher accelerates the impacting rod down a drop tower to impact the laterally extended moment arm of the helmet. The helmet is then constrained to coronal plane rotation. Characteristics of the helmet rotational acceleration profile (i.e., peak rotational acceleration and duration) are controlled by the mass and impacting velocity of the rod and mechanical characteristics of an elastomer placed at the impacting location on the helmet moment arm. All rotational acceleration profiles were developed and verified through our prior studies with this model and using post-mortem rats prior to *in vivo* testing for this study.

All injury and sham procedures were performed in the morning to allow a 6-h monitoring period after completion of the daily exposure, the single injury, or the sham protocols. Rats were individually transported to the laboratory and placed in an isoflurane induction box for 5 min. Once unconscious, rats were placed in the helmet, which was then attached to the test fixture. A nose cone was used to deliver isoflurane to the rat once inside the helmet and in the test fixture. The nose cone was removed just prior to head rotational acceleration exposure. The impacting rod was then accelerated down the drop tower to a pre-defined pre-impact velocity to impact the moment arm of the helmet and result in helmet/head rotational acceleration. Helmet rotational acceleration was measured using redundant linear accelerometers attached to the moment arm and high-speed video (5,000 frames per second) was used to verify the acceleration pulse and ensure minimal slippage of the helmet on the head. High and moderate exposure groups, which received thirty and eight head accelerations per day, respectively, received all daily accelerations consecutively during a single unconsciousness period. Subconcussive head rotational accelerations were delivered at a rate of approximately five head accelerations per minute. Rats receiving the sham procedure were exposed to all aspects of the experimental protocol, including placement in the helmet and test fixture, and anesthesia, without exposure to head rotational acceleration.

### Behavioral Assessments

**FIGURE 1:**
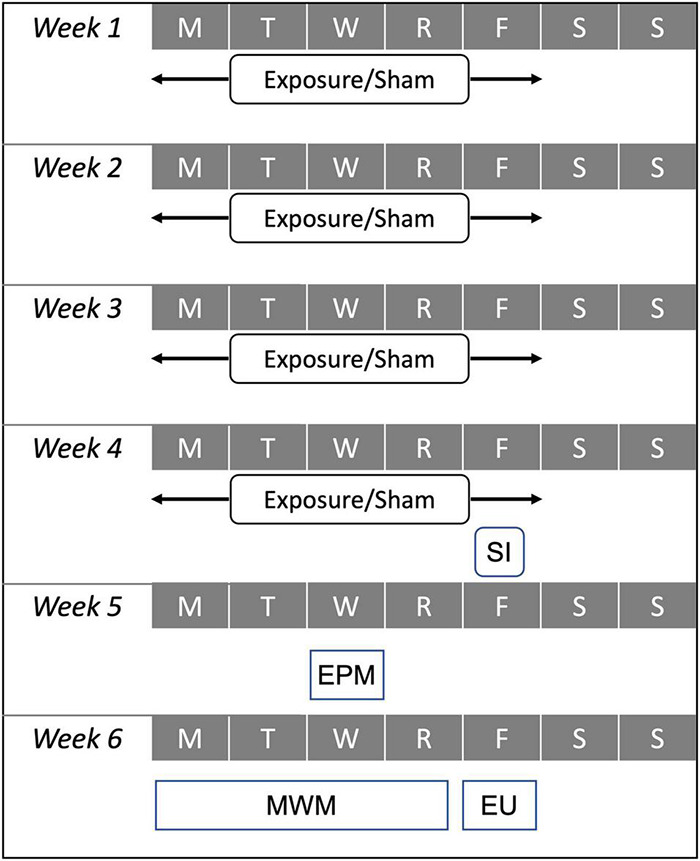
Experimental test matrix demonstrating relative timing for the 4-week exposure protocol used for high exposure (30 accelerations/day) and moderate exposure (eight accelerations per day) groups, the sham protocol, and the single injury (SI) exposure during the first 4 weeks of the protocol. Behavioral assessments including the elevated plus maze (EPM) and Morris water maze (MWM) were conducted during the fifth and sixth weeks of the protocol. All animals were euthanized (EU) on Friday of the sixth week of the protocol.

Behavioral assessments were conducted in the 2 weeks following the exposure protocol, the sham protocol, or the single injury procedure (Figure 1). The elevated plus maze (EPM) assessment was conducted to identify activity and emotional-type behaviors during the first post-injury week and the Morris water maze (MWM) was conducted to identify post-traumatic anterograde amnesia and spatial learning during the second post-injury week.

The elevated plus maze was located in its own sub-room within our behavioral testing laboratory and has four perpendicular 10-cm wide by 50-cm long arms suspended 82 cm above the floor. A 10-cm by 10-cm central platform connects the arms. One pair of opposing arms is enclosed by 32-cm high walls, while the other two arms and the center platform are not covered. The room has no external visual cues and is lighted independently from the general laboratory lights. At the beginning of the assessment, rats were placed on the center platform facing one of the two open arms. All rats were placed to initially face the same open arm. After placing the rats in the EPM, the laboratory technician left the room and monitored the animal’s activity from a separate workstation outside the behavioral assessment laboratory. The rats were allowed to freely explore the maze for 5 min and tracked using a digital video camera mounted above the maze. Noldus Ethovision software (Noldus Information Technology, Wageningen, Netherlands) was used to track and quantify the rats movements within the maze. Metrics quantified during the test included total distance traveled, total number of arm changes, total entries into and time spent in the open areas of the maze, and total entries and time spent in the closed arms of the maze. These metrics were quantified on a minute-by-minute basis across the 5 min of the assessment. Behaviors associated with post-injury activity included total number of arm changes and total distance traveled. Behaviors associated with changes in emotionality included entries into and time spent in the open and closed areas of the maze.

The 183-cm diameter Morris water maze was located in our behavioral testing laboratory. The MWM was filled with water to a depth of 25 cm and the water temperature was maintained within 1°C of 24°C. The testing paradigm consisted of 4 days of testing during the second post-injury week. Each testing day consisted of four trials wherein the rats were placed into the MWM facing the wall and allowed to explore the maze until finding and mounting a hidden 10-cm diameter platform with surface located 1-cm below the surface of the water. Rats were guided to the platform by the technician if they had not found the platform during the first 120 s of each trial in the maze. The platform was located in the same location for all trials, between the cardinal axes (i.e., SE) halfway along the radius between the center of the maze and the outer wall. Rats were placed into the maze at one of the four cardinal locations (N, E, S, and W) for each trial, in a different order for each day of testing. The MWM had numerous internal and external cues that the rats could use to help locate the hidden platform. A stationary video camera mounted above the maze was used to record the rat’s movements within the maze and Ethovision software was used to quantify a variety of metrics. Latency to find the hidden platform (s) was measured for each trial and was the primary metric used in this analysis. Total path length and swim speed were also quantified for each trial.

Statistical analysis focused on identifying groupwise differences in metrics quantified during the MWM and EPM assessments between the four experimental groups: high exposure, moderate exposure, single injury, and sham. MWM metrics (latency and path length) were analyzed using two-factor analysis of variance (ANOVA) with experimental group and day as the independent factors. Trials 2–4 for each MWM testing day were included in the analysis due to the inordinately high unsuccessful rate for Trial 1 on each testing day relative to the other three trials. EPM metrics were analyzed using two-factor ANOVA with minute and experimental group as the independent factors. Statistical analysis of behavioral data was performed using Stata version 12.1 (StataCorp, College Station, TX, United States).

### Blood Biomarker Analysis

Blood samples were obtained from the ventral tail artery (approximately 1 ml) of anesthetized rats for biomarker analysis. For both exposure groups, a total of six blood draws were obtained. The first blood draw occurred prior to the head acceleration exposure protocol (baseline). Additional blood draws were obtained on each Friday during the 4-week head acceleration exposure protocol after all head accelerations had been completed for the day. The last blood draw was obtained on Friday at the end of the 2-week behavioral protocol (terminal). For the single injury group, blood draws were obtained 1 week prior to the single injury procedure (baseline), immediately after the single injury, and at the end of the 2-week behavioral protocol (terminal). The sham group received a blood draw prior to the initiation of the sham protocol (baseline), on the last day of the 4-week sham protocol, and at the end of the 2-week behavioral protocol (terminal). All blood samples were placed in a microcentrifuge tube and allowed to clot at room temperature for 60 min before being microcentrifuged at 5,000 × *g* for 10 min. The serum supernatant (approximately 0.5 ml) was collected, snap frozen in liquid nitrogen, and stored at −80°C. Serum samples were shipped over night in containers of dry ice to our collaborators for analysis (Gryphon Bio).

Serum was analyzed for neurofilament light protein (NFL) concentrations. A “sandwich” ELISA was employed to detect the biomarkers of interest in the analyzed serum. Our procedure used the quanterix NF-light serum ELISA (research use only) from Quanterix 20-8002. Samples were used in 1/8 dilution which is a two-fold, according to the manufacturer’s instructions. Thus, our lower limit of detection (LLOD) was 0.2 pg/mL and lower limit of quantification (LLOQ) was 0.4 pg/ML (%CV < 6% intraassay and <12% interassay). Specifically, the assay was performed as follows. A sample of the serum was applied to one or more wells on a 96-well plate which had been coated with antibody (“capture antibody”) specific to the biomarker of interest. Any biomarker present in the serum was recognized by this antibody and thus bound to the well of the plate. The plate was washed to remove unbound (non-target) material. A second biomarker-specific antibody (“detection antibody”) was then added, which created a “sandwich” (the biomarker was sandwiched between two specific antibodies). The plate was again washed to remove any unbound detection antibody. The final stage was the readout step. This involved the introduction of a substrate that reacted with an enzyme (typically added in advance to the detection antibody) to produce a colored or chemiluminescent signal whose intensity was measured electronically and was directly proportional to the amount of detection antibody (and thus biomarker) present.

Determination of the biomarker concentration in a serum sample was performed by comparing the signal intensity from the unknown sample to a standard curve created from a series of calibrators of known biomarker concentrations run on the same 96-well plate as the unknown sample. Serum samples were generally tested using the same volume and under the same conditions as the calibrators. However, dilution of the serum was sometimes necessary in the case of an analyte concentration above the upper limit of quantitation of the assay or where the serum volume was insufficient to perform the test. In both cases, the adjusted concentration of the biomarker was reported and the dilution factor was noted. Quality control samples, consisting of a known concentration of biomarker in a representative matrix, were included on each plate to allow for confirmation of expected assay performance.

Groupwise comparisons in NFL concentration were analyzed using ANOVA techniques. Statistical analysis of blood biomarker data was performed using Stata version 12.1 (StataCorp, College Station, TX, United States). NFL concentrations were compared between the four groups at baseline, prior to the initiation of the exposure or sham procedures, or the single injury. Within group analyses were conducted to determine changes in serum concentration of NFL throughout the exposure protocol. Week-by-week groupwise comparisons were made between high and moderate exposure groups during the 4-week exposure protocol. Additionally, post-injury groupwise comparisons were made between the week 4 levels for the high and moderate exposure groups, and sham group, and the single injury post-injury time point. Finally, groupwise comparisons were made between all four groups at the terminal time point, which represented 2 weeks after the conclusion of the exposure and sham protocols, and 2 weeks after the single injury.

### Tissue Preparation, Immunohistochemistry, and Image Analysis

Rats were deeply anesthetized with Euthasol and transcardially perfused with PBS followed by buffered formalin. Brains were removed, post-fixed in formalin for 24 h, then immersed in 30% sucrose at 4°C until sectioning. Brains were cryosectioned (30 μm), and were prepared and stored in cryoprotectant (30% ethylene glycol, 30% glycerol in 0.02 M phosphate buffer). Free-floating sections were washed in PBS (0.01 M), then blocked in 1% BSA/0.4% Triton X-100/PBS for 1 h. Sections were incubated in primary antibodies (goat-α-Iba-1 1:1000, Abcam ab5076; rabbit-α-GFAP 1:2000, Abcam ab7260) in blocking solution for 48 h at 4°C. Next, tissue was rinsed in PBS and incubated in secondary antibodies (Donkey-α-Goat-Cy3 1:500, Jackson ImmunoResearch 705-166-147; Donkey-α-Rabbit-Alexa 488 Jackson ImmunoResearch 711-546-152) in blocking solution for 90 min at room temperature. Sections were then rinsed with PBS, mounted on slides, and coverslipped with Vectashield mounting medium (Vector Laboratories).

A Leica SP8 confocal microscope was used to capture images using a 25X/0.95 NA water objective at a 1024 × 1024 resolution. A z stack of 5 μm was collected, images were imported into Imaris 9.0.2 (Oxford Instruments), and slices were individually observed for any artifacts or tissue damage. Slices that were damaged were removed from analyses. Imaris spots, surfaces, and filaments algorithms were used for cell counts, surface area detection, and filament analysis. The spots parameter algorithm for DAPI cell count was calculated using an estimated diameter size of 5 μm, background subtraction set to ON, and quality set above 10.0. Surface parameters for glial fibrillary acidic protein (GFAP) was set at surface detail of 0.866 μm, background subtraction of 40 μm, manual threshold of 13.3, and the number of voxels set to above 30. Spots parameter for Iba1 + microglia/monocyte (M/M) cell count was calculated using an estimated diameter size of 8.00 μm, background subtraction set to ON, and quality set above 14.7. The surfaces parameters for the Iba1 + cells were set to a surface detail of 0.50 μm and a background subtraction of 40 μm with a threshold set at 12.2. Filament analysis for Iba1 + M/M was performed with the following parameters: Dendrite starting point diameter of 15 μm, seed point diameter of 1.3 μm, seed point threshold of 40, diameter around starting point of 15 μm, smooth width of 1.3 μm, remove disconnected segments threshold of 5, and maximum gap length of 6 μm.

Statistical analyses were performed by one-way ANOVA followed by Dunnett’s *post hoc* comparisons. In the case of a non-normal dataset (determined by D’Agostino-Pearson test), statistical analysis was performed by Kruskal–Wallis test followed by Dunn’s *post hoc* comparisons.

## Results

A total of 60 rats were used across the four groups in this study (*n* = 15 per group). All rats survived the single injury, exposure or sham procedures and were used for behavioral assessments, blood biomarker analysis, and immunohistochemistry.

### Elevated Plus Maze

All rats received a single 5-min exposure to the elevated plus maze (EPM) 5 days following the completion of the head acceleration exposure protocol, the sham protocol, or the single injury exposure. EPM behaviors were analyzed on a minute-by-minute basis. All experimental groups demonstrated a main effect of decreasing activity during the 5-min exposure to the EPM (*p* < 0.0001) (Table 1). Main effect groupwise differences were evident for the number of open area entries (*p* = 0.0420), total time spent in open areas (*p* = 0.0480), total time spent in the closed arms (*p* = 0.0466), and time spent in the closed arms per entry (*p* = 0.0479). A non-significant trend was also evident for total number of arm changes (*p* = 0.0897). These statistically significant differences were driven primarily by groupwise differences in activity during the second minute of the EPM assessment (Table 2). In general, high exposure rats demonstrated less activity and spent more time in the closed arms per entry during the second minute of the EPM assessment than shams and other groups. For example, high exposure rats had greater closed arm time despite fewer entries into the closed arms, which resulted in greater closed arm time per entry. Pairwise comparisons revealed that high exposure rats had significantly more closed arm time per entry than the single injury group (*p* = 0.007). High exposure rats also spent less time in the open areas than the single injury and moderate exposure groups, and had fewer entries into the open areas than all groups, although pairwise comparisons were not significant. Unlike some of the other metrics presented below, there was not a dose-dependent relationship between head acceleration exposures and EPM behaviors as the moderate exposure group often displayed unique behaviors compared to the high exposure group. However, similar to our previous findings (Stemper et al., 2016a) the single injury group demonstrated a more active response in the open areas of the EPM with more open area time than all other groups, more open area time per entry than all other groups, and more open area entries than all groups except the moderate exposure group.

**TABLE 1:** Distance traveled (cm) for each experimental group over the 5 min of the EPM assessment.

	Minute 1	Minute 2	Minute 3	Minute 4	Minute 5
Sham	311 ± 75 cm	287 ± 46 cm	213 ± 61 cm	192 ± 50 cm	199 ± 56 cm
Moderate exposure	345 ± 55 cm	305 ± 60 cm	218 ± 77 cm	230 ± 72 cm	169 ± 77 cm
High exposure	309 ± 64 cm	254 ± 76 cm	233 ± 66 cm	202 ± 77 cm	173 ± 58 cm
Single injury	304 ± 45 cm	285 ± 59 cm	239 ± 57 cm	223 ± 53 cm	198 ± 57 cm

*Total distance traveled significantly decreased (p < 0.0001) over the 5 min of the EPM assessment.*

**TABLE 2:** Open and closed arm metrics for each minute of the elevated plus maze assessment (mean ± standard deviation).

Behavior	Minute	*p*-value	Sham	Moderate exposure	High exposure	Single injury
Number	–	–	15	15	15	15
Total arm changes	1	0.5704	12.7 ± 3.0	13.3 ± 5.1	14.7 ± 4.7	13.9 ± 3.0
	2	0.1013	7.2 ± 3.5	9.6 ± 3.3	6.3 ± 4.8	8.5 ± 3.2
	3	0.4075	5.4 ± 3.0	6.3 ± 3.9	5.9 ± 2.2	7.2 ± 2.5
	4	0.6071	5.3 ± 3.2	6.5 ± 4.2	5.5 ± 3.2	6.9 ± 4.4
	5	0.4511	5.1 ± 2.6	4.3 ± 3.0	5.1 ± 3.5	6.2 ± 3.4
Closed arm time (s)	1	0.9224	39.1 ± 10.2	38.6 ± 10.2	38.6 ± 7.6	36.8 ± 11.8
	2	**0.0442**	55.4 ± 3.8	50.8 ± 6.7	52.6 ± 9.1	47.4 ± 9.6
	3	0.4244	53.7 ± 8.0	50.6 ± 12.4	55.1 ± 4.4	51.3 ± 7.2
	4	0.2329	54.8 ± 5.7	48.7 ± 13.5	54.7 ± 7.1	52.8 ± 8.0
	5	0.8103	55.4 ± 7.1	54.1 ± 7.9	52.7 ± 8.9	53.9 ± 5.7
Closed arm entries	1	0.6604	4.3 ± 1.8	4.4 ± 1.6	4.8 ± 2.1	4.0 ± 1.4
	2	0.0584	3.6 ± 1.5	4.4 ± 1.5	2.9 ± 1.7	3.7 ± 1.3
	3	0.3142	2.9 ± 1.2	2.8 ± 1.7	3.1 ± 1.1	3.6 ± 1.1
	4	0.6381	2.9 ± 1.5	2.9 ± 1.1	2.8 ± 1.2	3.4 ± 1.8
	5	0.4665	2.8 ± 1.1	2.4 ± 1.3	2.6 ± 1.5	3.1 ± 1.2
Closed arm time per entry (s)	1	0.6849	11.0 ± 5.9	9.8 ± 4.0	9.1 ± 3.1	9.9 ± 4.1
	2	**0.0035**	17.9 ± 7.2	13.1 ± 5.8	29.2 ± 22.8	14.1 ± 5.8
	3	0.1866	22.6 ± 12.5	26.5 ± 19.6	20.4 ± 11.7	15.8 ± 6.5
	4	0.7575	26.2 ± 17.6	22.0 ± 16.7	25.1 ± 15.9	20.7 ± 13.2
	5	0.4197	24.8 ± 15.6	31.1 ± 19.4	28.0 ± 18.0	21.3 ± 12.9
Open area time (s)	1	0.9300	20.8 ± 10.3	21.3 ± 10.1	21.4 ± 7.6	23.1 ± 11.8
	2	**0.0442**	4.6 ± 3.8	9.2 ± 6.7	7.4 ± 9.1	12.6 ± 9.6
	3	0.4244	6.3 ± 8.0	9.4 ± 12.4	4.9 ± 4.4	8.7 ± 7.2
	4	0.2329	5.3 ± 5.7	11.3 ± 13.5	5.3 ± 7.1	7.2 ± 8.0
	5	0.8103	4.6 ± 7.1	5.9 ± 7.9	7.3 ± 8.9	6.1 ± 5.7
Open area entries	1	0.4287	8.4 ± 1.9	8.9 ± 3.6	9.9 ± 3.7	9.9 ± 2.9
	2	0.1882	3.6 ± 2.2	5.2 ± 2.3	3.5 ± 3.4	4.8 ± 2.3
	3	0.3883	2.5 ± 1.9	3.5 ± 2.9	2.8 ± 1.4	3.6 ± 1.7
	4	0.5246	2.5 ± 2.0	3.7 ± 3.4	2.7 ± 2.1	3.5 ± 2.7
	5	0.5130	2.3 ± 1.7	1.9 ± 1.8	2.5 ± 2.2	3.1 ± 2.6
Open area time per entry (s)	1	0.7331	2.4 ± 1.1	2.7 ± 1.9	2.4 ± 1.1	2.2 ± 0.8
	2	**0.0197**	1.2 ± 0.4	1.6 ± 0.8	2.0 ± 1.1	2.5 ± 1.7
	3	0.8146	1.9 ± 1.6	2.2 ± 2.0	1.7 ± 1.3	2.2 ± 1.5
	4	0.3498	2.2 ± 1.7	2.7 ± 2.0	1.7 ± 1.2	1.7 ± 1.3
	5	0.6054	1.5 ± 1.2	2.6 ± 2.2	2.3 ± 1.7	2.4 ± 3.2

*High exposure rats spent more time and more time per entry in the closed arms, whereas rats receiving single injury spent more time and more time per entry in the open arms. P-values presented for independent ANOVA analyses conducted at each minute with experimental group as the independent factor. Bold values represent statistically significant groupwise differences (p < 0.05).*

### Morris Water Maze

**FIGURE 2:**
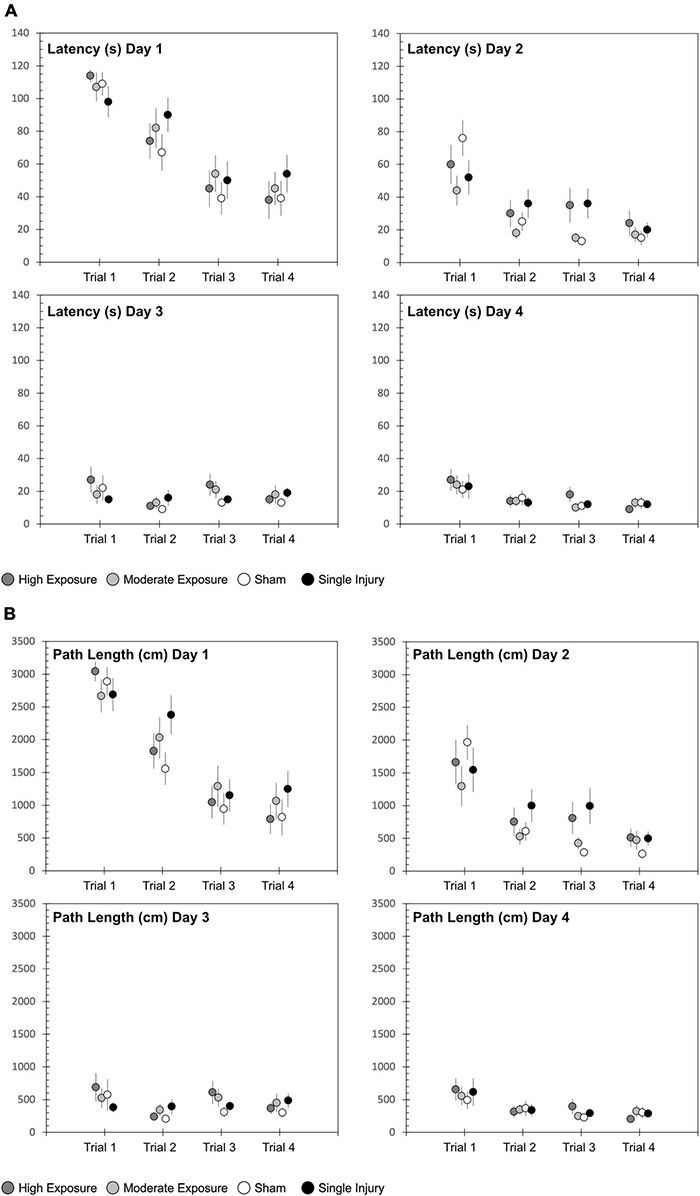
Morris water maze (MWM) **(A)** latency and **(B)** path length to find the hidden platform for each of the four experimental groups. The MWM protocol consisted of 4 days of four trials each, conducted over a 4-day period in the week following the exposure protocol, sham protocol, or single injury exposure. Data are presented as mean and standard error for of the four experimental groups.

Cognitive dysfunction was assessed using the Morris water maze. All rats received four consecutive days of testing that consisted of four trials per day. Groupwise differences in latency to find the hidden platform were quantified using two-factor ANOVA with independent factors experimental group and day. All groups demonstrated an inordinately high percentage of unsuccessful trials during the first trial of each day, so statistical analysis focused on latency to find the platform across the second, third, and fourth trials of each day. All groups demonstrated significant decreases in latency from the day 1 assessment to the day 4 assessment (*p* < 0.0001) (Figure 2A). Groupwise differences were evident for latency to find the platform (*p* = 0.0389). These differences were most evident during the second testing day, where pairwise comparisons revealed that high exposure rats had longer latencies to find the hidden platform than shams (*p* = 0.066). Likewise, single injury rats had significantly longer latencies to find the platform than shams (*p* = 0.004). Pairwise comparisons did not reveal any other statistically compelling groupwise differences in latency to find the hidden platform.

Similar findings were evident with regard to path length to find the hidden platform (Figure 2B). Path length decreased from day 1 to day 4 (*p* < 0.0001). Similar to latency, path length also demonstrated groupwise differences (*p* = 0.0025). That finding was primarily driven by groupwise differences on Day 2 of the MWM assessment, where high exposure rats (*p* = 0.0530) and single injury rats (*p* < 0.001) had longer path lengths to find the platform than shams. Swim speed was significantly different between sessions for both high and moderate exposure groups (high exposure: *p* = 0.0359; moderate exposure: *p* = 0.0103), but not the single injury group (*p* = 0.1781) or shams (*p* = 0.1111). However, the relative differences in swim speeds were minimal, with only 8% difference in swim speed between the slowest and fastest swim speeds for the high exposure group and only 14% difference in swim speed between the slowest and fastest swim speeds in the moderate exposure group. There was also no association between swim speed and latency to find the hidden platform, with the slowest or second slowest swim speeds occurring in the last MWM session for high and moderate exposure groups, respectively. Some significant groupwise differences in swim speed between injury groups were evident for sessions 1 (*p* = 0.0165), 3 (*p* = 0.0005), and 4 (*p* = 0.0013). However, once again, there was no association between swim speed and latency to find the hidden platform as there were no differences in swim speed during MWM session 2, where statistically significant groupwise differences were identified in latency to find the hidden platform (above).

### Serum Levels of Neurofilament Light

**FIGURE 3:**
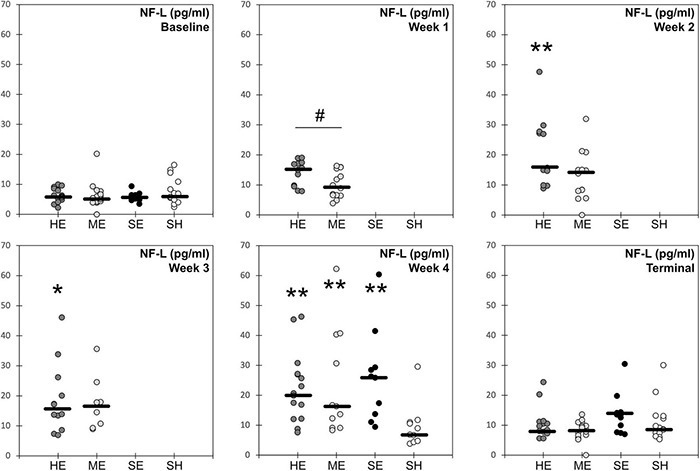
Blood serum concentration levels of neurofilament light protein for each of the four experimental groups (HE, high exposure; ME, moderate exposure; SE, single exposure; SH, sham). Solid horizontal lines represent the median groupwise data at each timepoint. * indicates statistically significant groupwise elevation relative to the baseline level for that experimental group. ** indicates statistically significant groupwise elevation relative to the baseline and terminal levels for that experimental group. # indicates significant difference in NFL concentrations between high and moderate exposure groups at that time point.

Blood samples obtained at multiple time points prior to and during the injury protocol for all experimental groups were analyzed for the serum concentrations of neurofilament light protein (NFL) (Figure 3). Our primary analysis focused on comparisons between high and moderate exposure groups. Two-factor ANOVA revealed statistically significant groupwise differences in serum NFL concentrations based on experimental group (*p* < 0.001) and blood draw time point (*p* < 0.001). *Post hoc* comparisons revealed that serum NFL concentrations for the high exposure group were elevated relative to baseline levels at the week 2 (*p* = 0.001), week 3 (*p* = 0.007), and week 4 (*p* < 0.001) blood draws. NFL concentrations in the high exposure group were also significantly elevated at the week 2 (*p* = 0.046) and week 4 blood draws relative to the terminal blood draw (*p* = 0.003), 2 weeks after the exposure protocol. Serum NFL concentrations for the moderate exposure group were elevated relative to baseline at the week 3 (*p* = 0.058) blood draw and significantly elevated relative to baseline at the week 4 (*p* < 0.001) blood draw. NFL concentrations in the moderate exposure group were also significantly elevated at the week 4 blood draw relative to the terminal blood draw (*p* < 0.001). These findings indicate differing temporal characteristics of serum NFL concentrations associated with the different exposure profiles.

Differences in serum NFL concentrations between experimental treatments were also evident for the two exposure groups. The high exposure group had significantly elevated NFL concentrations at the week 1 blood draw than the moderate exposure group (*p* = 0.032). NFL concentrations in the high exposure group were also elevated at the week 2 blood draw relative to the moderate exposure group, although the difference only approached statistical significance (*p* = 0.069). NFL concentrations between the high and moderate exposure groups were not different at baseline, weeks 3–4, or terminal blood draws. These groupwise differences indicate early elevation and plateau of serum NFL levels in rats receiving high numbers/frequency of head accelerations. However, serum NFL levels also increased with head acceleration exposures in the moderate exposure group, albeit at a slower and more dose dependent rate, eventually achieving the same levels as the high exposure group after the third weeks of the exposure protocol.

Groupwise differences in serum NFL concentrations were also evident following week 4 of the protocol (*p* = 0.023). *Post hoc* analysis revealed that rats receiving a single high magnitude acceleration exposure had somewhat greater serum NFL concentrations than both high and moderate exposure rats, although these individual *post hoc* comparisons were not statistically significant. This comparison was made between the 24-h post single injury blood draw and the week 4 blood draws for both high and moderate exposure groups. The week 4 blood draw represented the end of the exposure protocol for both exposure high and moderate groups, which also had the highest serum NFL concentrations of any blood draw for each group. Median serum NFL concentrations for the single injury group were 26% greater than the high exposure group (*p* = 0.51) and 60% greater than the moderate exposure group (*p* = 0.71). However, mean values were much closer between the three groups, with mean serum NFL concentrations in the single injury group only 16% and 11% greater than the high and moderate groups, respectively. Nonetheless, these findings demonstrate that both moderate and high levels of repetitive subconcussive exposure eventually produced similar elevations of serum NFL concentrations compared to the single injury group. Our prior studies demonstrated that acceleration magnitudes used in this study for the single injury group were consistent with mild traumatic brain injury (Stemper et al., 2015). These findings also provided some evidence of a prolonged effect of single injury and high exposure in the analysis of serum NFL concentrations. While concentrations had decreased over the 2 weeks from the week 4 to terminal blood draws, where no head acceleration exposures were provided, serum NFL concentrations remained elevated relative to baseline levels at the terminal blood draw for both the single injury (+132%; *p* = 0.002) and high exposure (+55%; *p* = 0.018) groups. The study design was not able to provide information on whether this prolonged elevation in serum NFL concentration was chronic and would have existed beyond 2 weeks following the injury or exposure protocol, or transient and would have eventually returned to baseline levels. This remains a focus for future investigations. However, serum NFL concentrations for the moderate exposure and sham groups at the terminal blood draw were not significantly different from baseline.

### Immunohistochemistry

**FIGURE 4:**
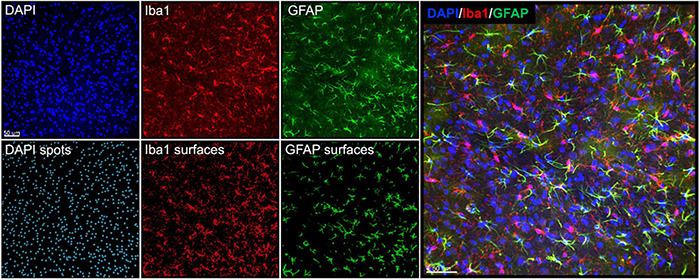
Representative images and Imaris renderings used in analyses. DAPI, Iba1, and GFAP were imaged from the same tissue as described in the section “Materials and Methods.” Spots were rendered from the DAPI channel, and surfaces were rendered from the Iba1 and GFAP channels to measure the total number of cells, number of Iba1 + cells, Iba1 intensity, and GFAP volume.

**FIGURE 5:**
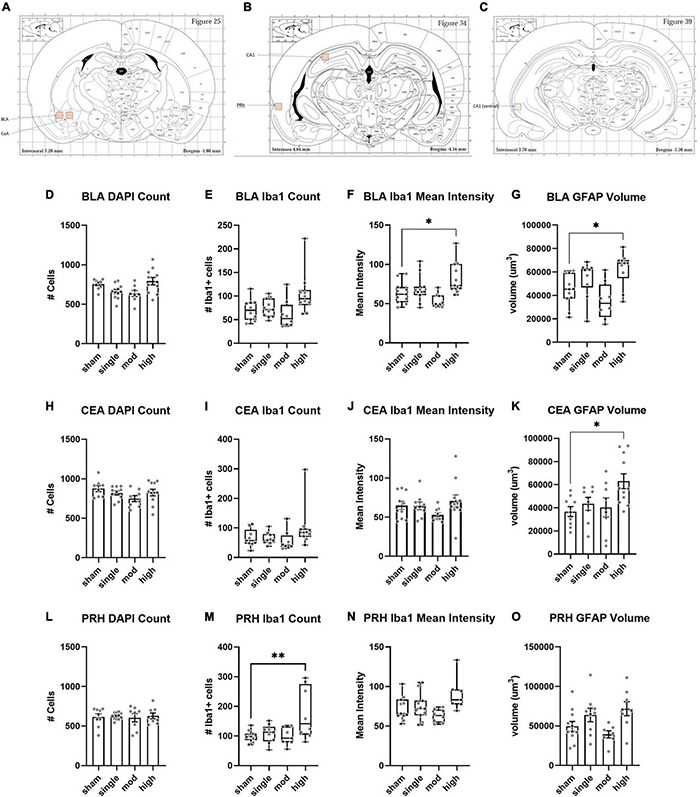
Histopathology analysis of amygdala and perirhinal cortex. **(A–C)** Regions of interests (ROIs) used for analyses. **(D–G)** Basolateral amygdala (BLA), **(H–K)** Central amygdala (CEA), **(L–O)** Perirhinal cortex (PRH). Bars represent mean ± SEM. Boxes/whiskers represent median, quartile, and range in non-normal datasets. Normally distributed data were analyzed by one-way ANOVA followed by Dunnett’s *post hoc* tests. Non-normal data were analyzed by Kruskal–Wallis tests followed by Dunn’s *post hoc* tests. *p ≤ 0.05, ^**^p ≤ 0.01.

We assessed histopathology 14 days after the completion of injuries. Our analysis was focused on previously identified injured brain regions following single rotational injury: the basolateral amygdala (BLA), central amygdala (CEA), perirhinal cortex (PRH), and CA1 hippocampus (Stemper et al., 2015). Outcome measures were the total number of cells (DAPI), the number of microglia/monocytes (M/M: Iba1 + cells), Iba1 mean intensity, and GFAP volume. DAPI was analyzed using the “spots” function in Imaris, which yielded the total number of nuclei stained. Iba1 and GFAP were analyzed using the “surfaces” function, and M/M morphology was analyzed using the “filaments” function (Figure 4). In the BLA, the total number of cells [*F*(3,35) = 4.3, η^2^ = 0.27, *p* = 0.011] and the number of M/M cells [H(3) = 9.9, η^2^ = 0.20, *p* = 0.020] were significantly different between groups, although *post hoc* testing did not find differences between the sham and any other groups in either of these measures (Figure 5). The mean intensity of Iba1 within identified surfaces (see section “Materials and Methods” for details) was also significantly different between groups [H(3) = 15.6, η^2^ = 0.36, *p* = 0.0014], with the high exposure significantly elevated over sham (η^2^ = 0.26, *p* = 0.049). The volume of GFAP+ surfaces was also altered by injury [H(3) = 14.8, η^2^ = 0.34, *p* = 0.0020], and the high exposure group had significantly greater volume than sham (η^2^ = 0.38, *p* = 0.014).

**FIGURE 6:**
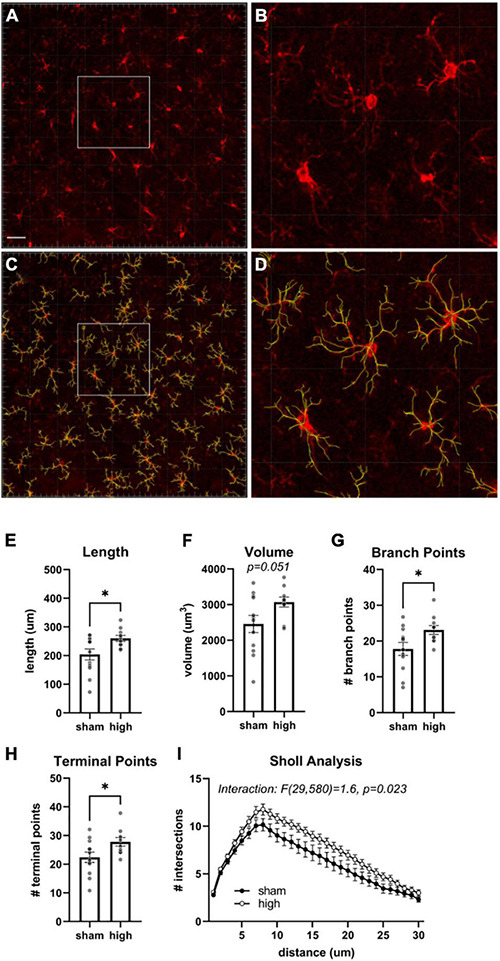
Morphological analysis of BLA microglia/monocytes. **(A)** Iba1 immunoreactivity. **(B)** Same image as **(A)** overlayed with rendered filaments. Scale bars = 50 μm. **(C,D)** Higher magnification views of depicted regions in **(A,B)**. Mean total length **(E)**, volume **(F)**, number of branch points **(G)**, and terminal points **(H)** of filaments generated from Iba immunostaining in the basolateral amygdala. **(I)** Sholl analysis of M/M identified by filaments analysis. Bars represent mean ± SEM. *p ≤ 0.05.

Microgliosis is associated with elevated levels of Iba1 and altered cellular morphology (Jacobowitz et al., 2012). Since BLA Iba1 levels were elevated in the high exposure group, we performed additional analyses in this region to determine if there was morphological evidence of altered M/M function following high exposure conditions. These analyses were performed using the Imaris “filament” function to trace patterns of Iba1 immunostaining (Figure 6). The mean filament length [*t*(16.9) = 2.6, η^2^ = 0.28, *p* = 0.020], number of branch points [*t*(20) = 2.3, η^2^ = 0.21, *p* = 0.030], and number of terminal points [*t*(20) = 2.3, η^2^ = 0.21, *p* = 0.034] were all significantly increased in high exposure rats. There was also a strong trend for increased filament volume [*t*(20) = 2.1, η^2^ = 0.18, *p* = 0.051] in the high exposure group. Next, we performed Sholl analysis to measure morphology at different distances from the M/M soma (Figure 6). There was a trend for a main effect of injury on Sholl intersections [*F*(1,20) = 3.9, *p* = 0.062] and a significant interaction effect [*F*(29,580) = 1.6, *p* = 0.023] between injury and distance from soma. Together, these data indicate that high rotational TBI exposure led to hyper-ramification of M/M cells in the BLA. This increase in morphological complexity of M/M cells has been observed following stress (Walker et al., 2013; Smith et al., 2019) and following diffuse brain injury or lesion of serotonergic neurons (Wilson and Molliver, 1994; Morrison et al., 2017).

The CEA was less affected by injury. The total number of cells was not affected by injury [*F*(3,33) = 1.7, η^2^ = 0.13, *p* = 0.19], and there was only a trend for a change in the number of Iba1 + cells [H(3) = 7.0, η^2^ = 0.13, *p* = 0.071] (Figure 5). Iba1 intensity was also not significantly affected by injury [H(3) = 6.6, η^2^ = 0.10, *p* = 0.088], however, the GFAP + volume was changed [*F*(3,32) = 3.9, η^2^ = 0.27, *p* = 0.017]. As observed in the BLA, GFAP volume was significantly elevated in the high exposure group relative to sham (η^2^ = 0.37, *p* = 0.011). The PRH showed different patterns of change following injury. The total number of cells was not different between groups [*F*(3,32) = 0.09, η^2^ = 0.008, *p* = 0.97], but the number of Iba1 + cells was [*F*(3,31) = 5.1, η^2^ = 0.33, *p* = 0.0055]. This difference was largely due to an increase in high exposure rats compared to shams (η^2^ = 0.33, *p* = 0.0042). There was also a significant difference between injury groups in Iba1 intensity [H(3) = 9.2, η^2^ = 0.19, *p* = 0.026], although none of the groups were significantly different from shams in *post hoc* comparisons (all η^2^ ≤ 0.21, *p* ≥ 0.15). GFAP volume was also different between groups in the PRH [*F*(3,31) = 3.5, η^2^ = 0.25, *p* = 0.027], but none of the groups were significantly different from shams (all η^2^ ≤ 0.21, *p* ≥ 0.09).

**FIGURE 7:**
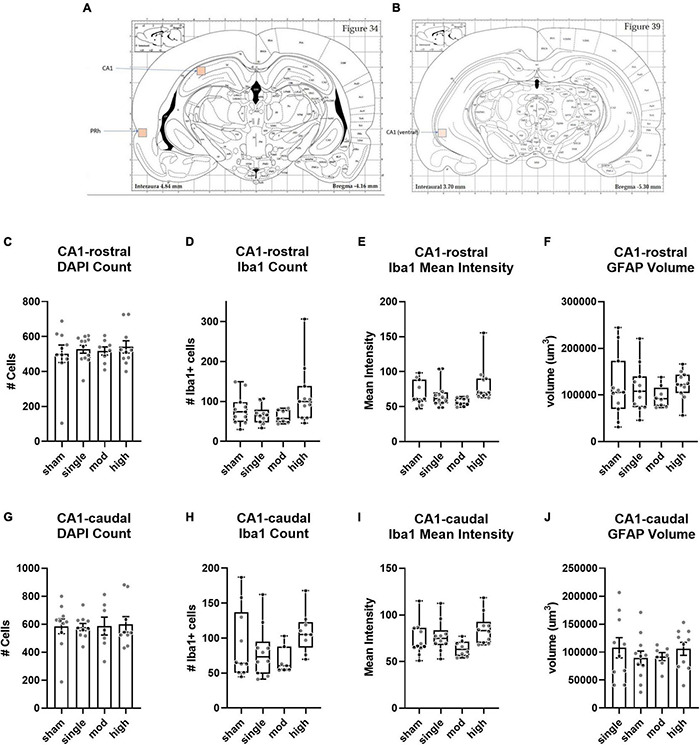
Histopathology analysis of CA1 hippocampus. **(A,B)** Regions of interest (ROIs) used for analyses. **(C–F)** Rostral/dorsal CA1, **(G–J)** Caudal/ventral CA1. Bars represent mean ± SEM. Boxes/whiskers represent median, quartile, and range in non-normal datasets.

Compared to the BLA and CEA subregions of the amygdala, there were more limited effects of injury on the hippocampus (Figure 7). In the rostral/dorsal CA1, there was no difference in total cell count [H(3) = 0.15, η^2^ = 0.079, *p* = 0.99] or number of Iba1 + cells [H(3) = 6.4, η^2^ = 0.097, *p* = 0.094]. Injury groups did differ in Iba1 intensity [H(3) = 11.2, η^2^ = 0.22, *p* = 0.011], but there was no effect of injury on GFAP volume [H(3) = 3.3, η^2^ = 0.008, *p* = 0.35]. The caudal/ventral CA1 showed a very similar pattern as the rostral/dorsal CA1. Neither total cells [*F*(3,32) = 0.026, η^2^ = 0.0024, *p* = 0.99] nor number of Iba1 cells [H(3) = 6.94, η^2^ = 0.12, *p* = 0.074] differed between groups. As observed in the rostral/dorsal CA1, Iba1 intensity differed between groups [H(3) = 8.9, η^2^ = 0.17, *p* = 0.031]. In both CA1 subregions, the high exposure group was most different from sham, although this did not reach statistical significance (η^2^ ≤ 0.15, *p* ≥ 0.17 for both subregions). As in the rostral/dorsal CA1, injury groups were not different in GFAP volume [*F*(3,34) = 0.048, η^2^ = 0.04, *p* = 0.70].

## Discussion

Human studies incorporating head impact measurement demonstrated that contact sport athletes can sustain tens to hundreds of head impacts per season in soccer (Lynall et al., 2016; Reynolds et al., 2017), ice hockey (Brainard et al., 2012; Mihalik et al., 2020), football (Crisco et al., 2010; Broglio et al., 2011; Stemper et al., 2019a; McCrea et al., 2021), and other sports. This level of repetitive subconcussive head impact exposure produced significant clinical and MRI changes in contact sports athletes with high levels of HIE, even in the absence of concussion (McAllister et al., 2012, 2014; Bazarian et al., 2014; Talavage et al., 2014). Repetitive head impact exposure has also been hypothesized to be a mechanism of incident concussion, (Talavage et al., 2014; Stemper et al., 2019b) although a direct causative relationship cannot be obtained from human studies. However, to date, preclinical studies have not modeled this type of exposure as prior efforts incorporated a limited number of exposures (Shultz et al., 2012b; Gangolli et al., 2019; Bree et al., 2020) or models with biomechanics not translatable to the human (Shultz et al., 2012b; Lavender et al., 2020). Modeling the characteristics of human head impact exposure in terms of the number, magnitude, and frequency of head accelerations is essential to understanding biomechanical mechanisms, characterizing behavioral disruptions, and identifying pathological processes associated with sport-related HIE and concussion. Incorporation of head rotational acceleration as the injury mechanism in our preclinical model enables the scaling and modeling of human head impact exposure in the rat. This study scaled subconcussive head accelerations from the human to the rat and exposed rats to a total of 160 or 600 head accelerations over the course of 4 weeks, which is representative of moderate and high levels of HIE for NCAA Division I football athletes during the fall preseason, a time of elevated concussion risk (McCrea et al., 2021).

Results of this preliminary study demonstrated that repeated exposure to very low-level head rotational accelerations produced behavioral and pathological changes in the rat. From a behavioral standpoint, rats receiving a high number of head acceleration exposures demonstrated statistically significant cognitive deficits with longer latencies to find the hidden platform and a less efficient search strategy in the MWM assessment. High exposure rats also demonstrated anxiety-like behaviors in the EPM with significantly more time per entry in the closed arms and decreased overall activity. Cognitive deficits in the high exposure group were consistent with the single injury group with similar latencies in the seventh overall trial of the MWM that were much greater than shams and the moderate injury group. However, results of the EPM assessment were different between the high exposure and single injury groups. Whereas high exposure rats had less activity and spent more time per entry in the closed arms of the EPM, the single injury group had increased activity, decreased time per entry in the closed arms, and increased time per entry into the open areas. Divergent behavioral outcomes between high exposure and single injury rats implies different injuries/mechanisms, and highlights the importance of this preclinical model in the understanding of concussive injury resulting from repeated subconcussive head acceleration exposures.

**FIGURE 8:**
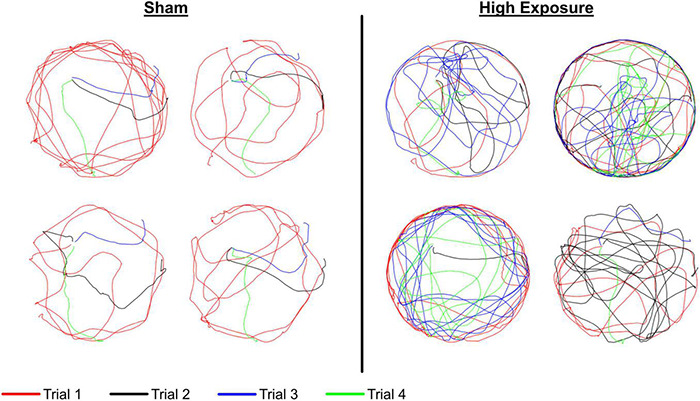
Representative swim paths for day 2 of the MWM protocol for sham **(left)** and high exposure **(right)** groups. Each of the four trials are represented and the platform was placed in the northwest **(upper left)** quadrant of the MWM for all four trials. These figures demonstrate a less effective search strategy for high exposure rats during trial 3 (blue), wherein a majority of sham rats found the platform during their initial pass and most high exposure rats either missed the platform or continued to search around the outside of the maze despite either finding or being shown the location of the hidden platform on two prior trials that day and four trials the previous day.

Increased MWM latency in high exposure and single injury rats was associated to a larger degree with an inefficient search strategy that was likely the result of decreased spatial learning. For example, eight sham rats found the hidden platform during their initial pass for day 2, trial 3, whereas only four high exposure rats found the hidden platform during their initial pass (Figure 8). Additionally, after a less focused search strategy during the first trial of day 2, sham rats were more likely to recall the platform position and find the hidden platform more efficiently on subsequent trials during day 2. However, fewer high exposure rats demonstrated that level of spatial learning and a majority continued to demonstrate less focused search strategies in the second, third, and fourth trials of day 2. These subtle differences in cognitive abilities between high exposure and sham rats may be indicative of developing cognitive dysfunction associated with high levels of repetitive subconcussive exposures and is consistent with human studies on non-concussed athletes that demonstrated cognitive changes in athletes with high HIE (McAllister et al., 2012). Analysis of serum levels of NFL also demonstrated remarkable findings. Elevated serum levels of NFL are indicative of axonal damage in the brain (Shahim et al., 2016). NFL has shown promise as a prognostic biomarker for axonal damage in sport-related concussion. For example, cerebrospinal fluid (CSF) levels of NFL were correlated to the severity of brain injury in boxers (Zetterberg et al., 2006). Serum NFL levels were also shown to be elevated following concussion in Australian football athletes (McDonald et al., 2021) and following a single season of head impact exposure in non-concussed American football athletes (Oliver et al., 2016). In the current study, blood serum levels of NFL protein in the high exposure group were significantly elevated relative to baseline at the weeks 2, 3, and 4 blood draws (*p* < 0.05 at each time point). Serum levels of NFL were also elevated relative to baseline in the moderate exposure group at the week 3 (*p* = 0.058) and week 4 (*p* = 0.003) blood draws. Similar elevation in NFL concentration was also evident following single high magnitude head acceleration (*p* = 0.011). More interesting, however, was that NFL levels demonstrated a graded response to head acceleration exposure throughout the course of the exposure protocol. Both high and moderate exposure groups demonstrated incremental changes in serum NFL levels for each of the 4 weeks of the exposure protocol. High exposure rats demonstrated a more dramatic increase relative to moderate exposure rats during the first 2 weeks, with significantly greater serum NFL levels at the week 1 blood draw (*p* = 0.032) and a trend of greater NFL levels at the week 2 blood draw (*p* = 0.069). This finding implies greater axonal damage with higher frequency exposures (150 accelerations per week) in the high exposure group compared to lower frequency exposures in the moderate exposure group (40 accelerations per week). The clinical implication of these findings are strong in that reduced head impact exposure likely reduces cumulative axonal damage associated with repetitive subconcussive exposures. From an injury prevention standpoint, this finding would indicate that reducing head impact exposure during contact practices or reducing the frequency of contact practices could significantly reduce the risk of concussion for contact sport athletes.

Our histopathology measurements were guided by a prior neuroimaging study, which found that the BLA, CEA, hippocampus, and associated cortices were susceptible to injury in the rotational TBI model employed here (Stemper et al., 2015). We found that the BLA was the most affected by injury, reflected by increases in Iba1 intensity and GFAP volume in the high exposure animals. Both markers are associated with TBI-induced reactive gliosis (Mouzon et al., 2012; Acaz-Fonseca et al., 2015), and further analysis of the Iba1+ cells identified pronounced hypertrophy of the processes. This hyper-ramification has been associated with exposure to chronic stress and has been postulated to drive stress-associated changes in behavior (Walker et al., 2013; Hellwig et al., 2016). These histopathological findings mirror pairwise differences between the high exposure and sham groups identified in blood serum levels of NFL as well as evidence of cognitive deficiencies in the Morris water maze and anxiety-like behaviors in the elevated plus maze. Taken together, these findings provide evidence of pathological and behavioral changes associated with repeated subconcussive exposures.

This preclinical model for human-relevant repetitive subconcussive head acceleration exposure represents a unique and important scientific advancement and tool for the investigation of injury mechanisms associated with sport-related concussion. Prior research demonstrated the importance of head rotational acceleration in the traumatic brain injury mechanism and reported that greater head rotational accelerations were associated with a greater injury risk and more severe pathological outcomes (Gennarelli et al., 1972, 2003; Zhang et al., 2006). Incorporation of head rotational acceleration as the injury mechanism in this model makes the results and biomechanical tolerance information derived from the model directly scalable between the human and rodent (Panzer et al., 2014). For example, head impact exposure measured in human contact sport athletes can be scaled down to the rodent, as was done here, or rodents can be used to characterize injury tolerance data in terms of number, magnitude, and frequency of head accelerations that can be scaled up to the human. Investigation of head impact exposure in contact sport athletes have identified that athletes with a greater number of impacts, higher magnitude impacts, and more frequent impacts may be more susceptible to concussive injury (Broglio et al., 2017; Stemper et al., 2019b), however, the mechanism for increased injury risk is not clear. Preclinical models are ideal to characterize effects of subconcussive exposures in the injury mechanism as they remove confounding factors in humans including variability associated with genetic and concussion/head impact exposure history, as well as differences in propensity to report concussion based on individual and social characteristics. Determining human-relevant injury mechanisms and tolerance remains a focus of this ongoing research.

This study is unique as prior investigations focused on “subconcussive” exposures incorporated less biofidelic models with decreased relevance to the human biomechanical injury mechanism. For example, subconcussive lateral fluid percussion injury was defined according to fluid pressure magnitude, produced no behavioral changes, but resulted in an inflammatory response (Shultz et al., 2012b). However, the fluid percussion injury mechanism is not biofidelic to the human, recorded pressures are not translatable to the human, and therefore, the model is not well suited for the investigation of injury mechanisms from human contact sport head impact acceleration. Other studies focused on a weight drop mechanism that produced injury through head impact and acceleration. Bree incorporated three subconcussive exposures spaced 72 h apart (Bree et al., 2020) and Lavender used a more contact sport-relevant ten consecutive subconcussive exposures 3 days per week over 2–12 weeks (Lavender et al., 2020). While the mechanism of head impact leading to acceleration more closely models the human condition than lateral fluid percussion, head accelerations were not reported or based on human exposures, the initial impact may have been associated with some level of focal injury that is not seen in human concussion (Nilsson and Nordström, 2009), and impact acceleration stands the risk of rebound impacts (O’Connor et al., 2011). Alternatively, as demonstrated in immunohistochemistry findings reported in this manuscript and diffusion tensor imaging findings reported previously (Stemper et al., 2015), our head rotational acceleration model produces diffuse injury without focal pathology that is consistent with human injury outcomes. Therefore, this coronal plane head rotational acceleration model is ideal for the investigation of injury tolerance associated with repetitive subconcussive exposures.

One issue that needs further clarification prior to accurate quantification of human-relevant tolerance to repetitive subconcussive exposures is the relative timing of repeated exposures. As an initial estimate, this study exposed rats to the same frequency of exposures (i.e., number of accelerations per day) as we measured in contact sport athletes. However, frequency of exposures between rats and humans likely needs to be scaled based on differences in body size, metabolic rate, physiological time, and injury recovery profiles between species. Rat metabolic and inflammatory responses from instances of TBI are significantly faster than humans (Cox et al., 2006; Shitaka et al., 2011; Thelin et al., 2017). Another study indicated that TBI-related axonal injuries in humans match the response in rats during the acute phase (Agoston et al., 2017). In a review of neurotrauma scaling, Panzer indicated that relative metabolic rate between species can be approximately scaled by an exponent of 0.75 (Kleiber and Rogers, 1961; Hofman, 1983). Physiological time such as respiratory cycle, cardiac cycle, and life cycle can be approximately scaled by an exponent of 0.25 (Stahl, 1967; Lindstedt and Calder, 1981). However, the injury mechanism associated with repetitive subconcussive exposure is likely more complicated, with accumulating damage associated with each subinjury, secondary injury processes, recovery profile, and their interruption with subsequent head acceleration exposures. Faster metabolic and inflammatory responses in rats, as described above, likely means that the frequency and duration of head acceleration exposures used in this study had less of an effect on rats than it did on the humans that were used to determine these exposure characteristics. Accordingly, it would be likely that cognitive, emotional, and pathological changes identified in these rats may be enhanced in humans with the same exposure profile. This may be particularly true when considering that American football and other contact sport seasons last for months instead of 4 weeks as presented here. Literature has provided amble evidence of cognitive and MRI changes in non-concussed contact sport athletes that supports this assumption (McAllister et al., 2012, 2014; Bazarian et al., 2014; Talavage et al., 2014; Davenport et al., 2016; Montenigro et al., 2017). Understanding these changes in humans using a preclinical rodent model is dependent on accurate scaling relationships for acceleration magnitudes and relative exposure durations.

This manuscript presents a rodent model to study the mechanisms and tolerance for injury resulting from repetitive subconcussive head accelerations. The use of coronal plane acceleration allows for the scaling of daily human exposures, measured on field during routine participation in contact sports, to the rat in terms of the severity, number, and frequency. Results presented here highlight behavioral and pathological changes associated with repeated exposure to very low-level head rotational accelerations for the high exposure group. Cognitive changes identified in the Morris water maze mirror cognitive deficits identified in high exposure contact sport athletes following a single season without concussion (Talavage et al., 2014). Significant changes in emotionality identified in the elevated plus maze represent a novel finding that, to our knowledge, has not been investigated or reported in human studies. Perhaps even more interesting, histopathological and blood biomarker analyses, however, identified progressive gliosis and axonal damage associated with higher levels of subconcussive exposure, wherein significant differences in histopathological markers of gliosis were identified in the high exposure group. Differing profiles of axonal damage were identified between high and moderate exposure groups during the exposure protocol using blood biomarker analysis. The high exposure group demonstrated a profile more consistent with a step increase in serum NFL levels during the first week of the exposure protocol that increased only slightly during weeks 2–4. However, the moderate exposure group demonstrated a more linear and dose-dependent response with consistent increases in serum NFL levels during the first 3 weeks of the exposure protocol that was more indicative of accumulating axonal injury associated with moderate levels of repeated subconcussive exposures. These findings are important as they highlight the possible benefit from reducing head impact exposure in contact sport athletes. However, these findings represent only a first step in understanding the combined effects of head impact frequency, severity, and duration of subconcussive head accelerations on behavioral and pathological outcomes.

## Data Availability Statement

Data that support the findings of this study are available from the corresponding author upon reasonable request.

## Ethics Statement

The animal study was reviewed and approved by the Clement J. Zablocki Veterans Affairs Medical Center Institutional Animal Care and Use Committee (IACUC).

## Author Contributions

BDS, CO, KW, MB, and MM contributed to the conception of the study hypothesis and protocol. AS, RC, CM, KJ, BS, and JS contributed to the data collection. BDS, CO, AS, KW, MB, and MM contributed to data analysis and synthesis. BDS wrote the first draft of the manuscript. CO, AS, RC, CM, KJ, BS, JS, KW, MB, and MM contributed to revision and editing of the first draft of the manuscript. All authors approved the final submitted version of the manuscript.

## Conflict of Interest

KW was employed by the Gryphon Bio, Inc. The remaining authors declare that the research was conducted in the absence of any commercial or financial relationships that could be construed as a potential conflict of interest.

## Publisher’s Note

All claims expressed in this article are solely those of the authors and do not necessarily represent those of their affiliated organizations, or those of the publisher, the editors and the reviewers. Any product that may be evaluated in this article, or claim that may be made by its manufacturer, is not guaranteed or endorsed by the publisher.
